# Surgical management of primary and secondary pilocytic astrocytoma of the cerebellopontine angle (in adults and children) and review of the literature

**DOI:** 10.1007/s10143-020-01293-4

**Published:** 2020-04-15

**Authors:** Sasan Darius Adib, Martin U. Schuhmann, Johann-Martin Hempel, Antje Bornemann, Rocio Evangelista Zamora, Marcos Tatagiba

**Affiliations:** 1 Department of Neurosurgery, University of Tuebingen, Hoppe-Seyler-Str. 3, 72076 Tuebingen, Germany; 2 Department of Neuroradiology, University of Tuebingen, Hoppe-Seyler-Str. 3, 72076 Tuebingen, Germany; 3 Department of Neuropathology, University of Tuebingen, Hoppe-Seyler-Str. 3, 72076 Tuebingen, Germany

**Keywords:** Cerebellopontine angle, CPA, Pilocytic astrocytoma, Glioma, Brainstem, Facial nerve

## Abstract

Glial tumors in the cerebellopontine angle (CPA) are uncommon and comprise less than 1% of CPA tumors. We present four cases of pilocytic astrocytoma of the CPA (PA-CPA) that were treated in our department. Patients who received surgical treatment for PA-CPA from January 2004 to December 2019 were identified by a computer search of their files from the Department of Neurosurgery, Tübingen. Patients were evaluated for initial symptoms, pre- and postoperative facial nerve function and cochlear function, complications, and recurrence rate by reviewing surgical reports, patient documents, neuroradiological data, and follow-up data. We identified four patients with PA-CPA out of about 1500 CPA lesions (~ 0.2%), which were surgically treated in our department in the last 16 years. Of the four patients, three were male, and one was a female patient. Two were adults, and two were children (mean age 35 years). A gross total resection was achieved in three cases, and a subtotal resection was attained in one case. Two patients experienced a moderate facial palsy immediately after surgery (House–Brackmann grade III). In all cases, the facial function was intact or good (House–Brackmann grades I–II) at the long-term follow-up (mean follow-up 4.5 years). No mortality occurred during follow-up. Three of the patients had no recurrence at the latest follow-up (mean latest follow-up 4.5 years), while one patient had a slight recurrence. PA-CPA can be safely removed, and most complications immediately after surgery resolve in the long-term follow-up.

## Introduction

Pilocytic astrocytomas (PA) comprise 6% of all intracranial tumors and are the most (in some studies the second most [30]) common primary brain tumors in children. Over 40% [9] of PA are localized in the cerebellum, followed by supratentorial locations (35%) [9]. Other typical locations are the optic pathway and hypothalamus [30] (11%) [9], the brainstem (9%) [9], and the spinal cord.

Pilocytic astrocytomas of the cerebellopontine angle (PA-CPA) are rare [30] and might grow as primary PA (which have their origin in the root entry zone of cranial nerves in the CPA) [2, 16] or as exophytic brainstem or cerebellar PA with secondary invasion of the CPA [2, 16]. They may mimic other tumors such as vestibular schwannoma [37, 40, 48].

PA-CPA are a formidable challenge from neurosurgical perspective, and different strategies are necessary to protect surrounding neurovascular structures.

The goal of this retrospective study is to analyze the surgical management of exophytic PA of the brainstem with invasion of the CPA, especially with focus on the clinical presentation, surgical strategy, the extent of resection, and early and late treatment outcomes.

## Materials and methods

### Data collection and inclusion criteria

Patients who had undergone surgery for PA-CPA from January 2004 to December 2019 were identified through a computer search of the patients’ medical files at our neurosurgery department. Four patients with PA-CPA of > 1500 CPA lesions (0.2%) had undergone surgery in our department. The patients were evaluated for presenting symptoms, preoperative and postoperative facial nerve function (according to the House and Brackmann scale), cochlear function (according to the Gardner–Robertson scale), extent of resection, recurrence rate, survival rates, and complications by reviewing patient documents, surgical reports, neuroradiological data, and follow-up data. Furthermore, the surgical strategy was analyzed. PA-CPA had been confirmed histopathologically in each case.

The study was approved by the ethics committee.

### Surgical technique and strategies

Three patients had been positioned under general anesthesia in supine position and one in semisitting position according to our standards. The anesthesiologic setup in the case of semisitting position included transesophageal echocardiography for early detection of air emboli. All patients underwent a retrosigmoid craniectomy. Intraoperative monitoring included motor evoked potentials (MEP) and electromyography (EMG) recordings of the facial nerve and the lower cranial nerves, and auditory evoked potentials and MEP and sensory evoked potentials of the upper and lower extremities.

### Follow-up

Clinical examination for facial nerve (according to the House and Brackmann scale) and hearing function (according to the Gardner-Robertson scale) was performed in all cases 3 days after surgery and was repeated at the 3-month follow-up. Postoperative MRI-examinations were performed 3 months after surgery. Further follow-up examinations have been performed every 6 months for at least 1 year.

## Results

Four patients (three were male, and one was female) with PA-CPA were included in this study. Two were adults (ages 53 years and 71 years), and two were children (ages 7 years and 10 years). Three PA-CPA were located on the right side, and one was on the left side.

### Radiological findings

**Fig. 1:**
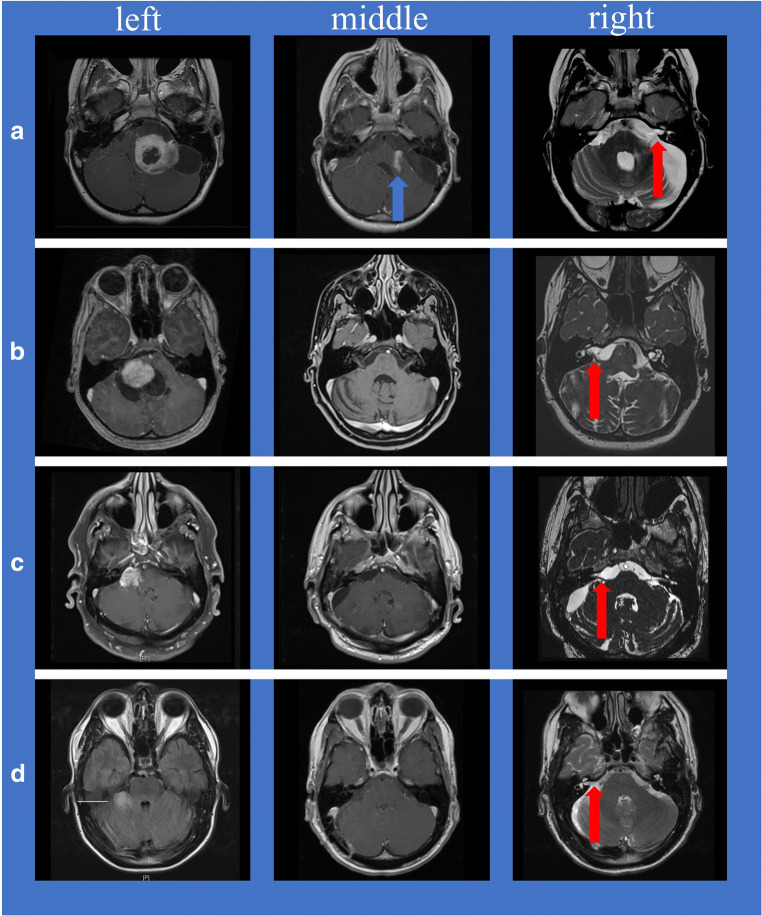
**a** Left (case 1), **b** left (case 2), **c** left (case 3), **d** left (case 4): Preoperative axial contrast-enhanced T1-weighted magnetic resonance imaging scans of each patient with PA-CPA (A+B: pediatric PA-CPA presented typically with a cystic cerebellar mass with enhancing mural nodule; C+D: adult PA-CPA presented atypically with only solid tumor components). **a** Middle, **b** middle, **c** middle, **d** middle: Postoperative axial contrast-enhanced T1-weighted magnetic resonance imaging scans; a gross total resection (GTR) of the PA-CPA was achieved in three cases (3/4). In one case, only a subtotal removal of the tumor was performed (blue block arrow). **a** Right, **b** right, **c** right, **d** right: Postoperative axial T2-weighted magnetic resonance images; anatomical facial nerve preservation was achieved during surgery in all cases (red block arrows)

Three patients had secondary PA-CPA arising from the cerebellum (1/4) and the cerebellar peduncle (2/4), and one patient had a primary PA-CPA (with arising from the root entry zone of the VIII cranial nerve). MRI exams showed cystic PA-CPA in two cases (Fig. 1a left and 1b left) and non-cystic PA-CPA in two cases (Fig. 1c left and 1d left). In the two cystic PA-CPA cases, the MRI revealed brainstem compression with displacement and secondary hydrocephalus. One of the non-cystic PA-CPA cases also showed moderate brainstem compression (Fig. 1c left). Both patients with cystic PA-CPA were children (mean age 8.5 years), whereas both patients with non-cystic PA-CPA were adults (mean age 62 years) (Table 1). The pediatric PA-CPA presented typically with a cystic cerebellar mass with enhancing mural nodule (Fig. 1a left and 1b left). Contrary, adult PA-CPA were atypical: both showed only solid tumor components (Fig. 1c left and 1d left). One adult PA-CPA showed no contrast enhancement (Fig. 1d left).

**Table 1:** Histopathological and radiological findings and further oncological therapy

	Age/sex	Histopathological confirmed	Cystic component	Brainstem compression	Extent of resection	Recurrence	Further oncological therapy
1	7/m	Yes	Yes	Yes	Subtotal	Slight progression	No
2	10/f	Yes	Yes	Yes	Total	No	No
3	71/m	Yes	No	Yes (moderate)	Total	No	No
4	53/m	Yes	No	No	Total	No	No

### Presenting symptoms

1. This 7-year-old male presented with slight facial palsy (H+B grade II), nausea, vomiting, and nystagmus for 2 months.
2. This 10-year-old female with a history of dyspnea for 2 years presented in our outpatient ambulance with slight facial palsy (H+B grade II), disturbance in walking, nausea, vomiting, and mild hemiparesis for 1 month.
3. This 71-year-old male presented with disturbance in walking, urinary incontinence, and slight dementia for many months.
4. This 53-year-old male with a history of a testicular carcinoma, which had been treated by surgery and chemotherapy, presented with disturbance in coordination and sensitivity of the left arm for 10 days.

All presenting symptoms were related to the compression of the brainstem or cranial nerves, or to hydrocephalus.

The most common symptoms were facial palsy (2/4) on the side of the PA-CPA (H+B II), disturbance in walking (2/4) [one of them was due to the compression of the brainstem, and one of them was due to hydrocephalus, including urinary incontinence (1/4) and slight dementia (1/4)], and vomiting and nausea (2/4).

Other symptoms included slight hemiparesis (1/4), disturbance of coordination and sensitivity of the left arm (1/4), dyspnea (1/4), and nystagmus (1/4).

### Tumor resection strategy

**Fig. 2:**
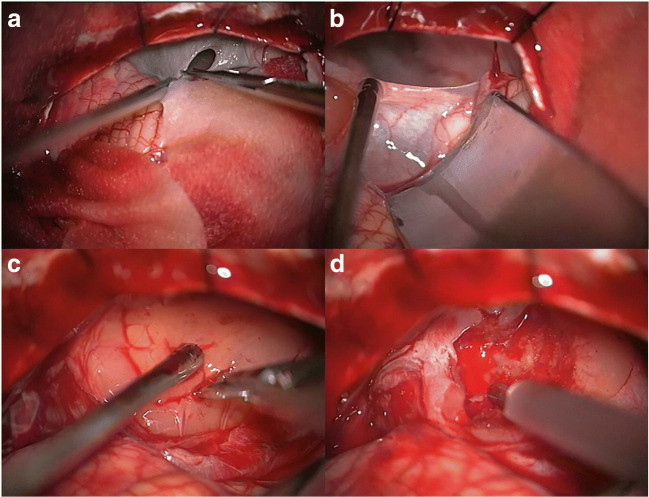
Different steps of tumor resection strategy: in case of large cystic component, a decompression of the cyst should be performed (**a**) and, furthermore, if possible, a removal of the cyst wall (**b**). After removal of the cyst, the surface of the solid tumor portion was incised (**c**) and a debulking was performed (**d**)

1. Cystic and non-cystic PA-CPA:

In case of large cystic PA-CPA (Fig. 1a left and 1b left) a decompression of the cyst should be in most cases the first step (Fig. 2a). The cystic wall should be removed (Fig. 2b), but only in case that it is not too adherent to surrounding structures. After removal of the cyst, the surface of the solid tumor portion was incised (Fig. 2c) and a debulking (using an ultrasonic aspirator) was performed (Fig. 2d). After debulking, the tumor was dissected from surrounding structures.

2. Primary and secondary PA-CPA:

In the case of primary PA-CPA (with arising from the root entry zone of the vestibular nerve), the vestibular nerve had to be sacrificed. In secondary PA-CPA, the extent of resection depends on the infiltration of the brainstem, cerebellar peduncle, and cerebellum (see below).

### Extent of resection and recurrence rate

A gross total resection (GTR) of the PA-CPA was achieved in three cases (3/4) (Table 1) (Fig. 1b middle, 1c middle, and 1d middle). In one patient (case 1), only a subtotal removal of the tumor was performed (Fig. 1a middle) to preserve the facial nerve function and because of infiltration of the cerebellar peduncle (Fig. 1a left and 1a middle).

Three of the patients had no recurrence at the latest follow-up (mean latest follow-up of all patients 4.5 years) (Table 1). Pediatric patient with incomplete resection of PA-CPA demonstrated slight progression of residual tumor tissue 1 year after resection. In the further follow-up, no further progression was observed.

### Histopathological results

PA was histologically confirmed in each case (Table 1) with typical characteristics of low cellularity, presence of Rosenthal fibers, eosinophilic granular bodies, and a biphasic consistency with microcystic and compact areas.

### Further therapies

None of the patients received radiotherapy or chemotherapy before or after surgery (Table 1). One patient had a VP shunt inserted during a second procedure due to the remaining hydrocephalus (case 3).

### Facial nerve function

Anatomical facial nerve preservation was achieved in all four patients (Fig. 1a right, 1b right, 1c right, and 1d right). Before surgery, two patients (case 1 and 2) had a slight facial palsy (H+B grade II) (Table 2). Immediately after surgery, two patients (case 1 and case 3) had a moderate facial palsy (H+B grade III), and one (case 2) had a slight facial palsy (H+B grade II) (Table 2).

**Table 2:** Preoperative and postoperative facial (H+B = House–Brackmann grade) and cochlear function

	Age/sex	Extent of resection	Preoperative facial function (H + B)	Facial function immediately after surgery (H+B)	Facial function in long term (H+B)	Preoperative cochlear function	Postoperative cochlear function
1	7/m	Subtotal	II	III	II	No hearing impairment	Surditas on one side
2	10/f	Total	II	II	I	No hearing impairment	No hearing impairment
3	71/m	Total	I	III	II	No hearing impairment	Surditas on one side
4	53/m	Total	I	I	I	No hearing impairment	No hearing impairment

In two of the four cases, no functional impairment of the facial nerve (H+B grade I) was detectable at the 3-month follow-up examination. In the two other cases (cases 1 and 3), there was a slight palsy of the facial nerve (H+B grade II) at the 3-month follow-up and long-term follow-up (Table 2).

### Cochlear nerve function

None of the patients had a cochlear dysfunction (GR grade I) before surgery (Table 2). Anatomical cochlear nerve preservation was achieved in three patients (all patients except the one with primary PA-CPA arising from VIII cranial nerve). Two patients (one with primary and one with secondary PA-CPA) had hearing loss (GR grade V) after surgery on the side of surgery (case 1 and case 3) (Table 2).

### Complications

Further complications (besides facial and cochlear nerve dysfunction) immediately after surgery included palsy of the VI (1/4) and XII (1/4) cranial nerves (case 2), dysphagia (1/4) (case 3), and hemiparesis (2/4) (in case 1 a new hemiparesis and in case 2 worsening of a previously known hemiparesis). All these symptoms recovered in the long term, except for a slight remnant hemiparesis in one case (case 2). No mortality occurred during follow-up.

## Discussion

Most common lesions in the CPA are vestibular schwannoma (70–80%) [4], meningioma (10–15%) [4], followed by epidermoid cysts (5%) [4]. The remaining percentage includes a variety of different lesions [4, 5].

CPA gliomas are rare [2, 16] and can be classified into primary and secondary CPA gliomas [2, 16].

### Primary CPA glioma

Primary CPA glioma arises from the root entry zone (either the glial segment or the transition zone) of the cranial nerves in the CPA [2, 16]. Cranial nerves comprise three distinct histologically segments: the glial segment, a transition zone, and a peripheral segment [2, 16].

The glial segment is histologically identical in structure to the central nervous system (the nerve axons are supported by neuroglia: astrocytes and oligodendrocytes), while the peripheral segment structure is similar to a peripheral nerve (nerve fibers are insulated by Schwann cells) [2, 16].

Between glial and peripheral segments lies a transitional or intermediate zone. Various studies analyzed the differences in length of different segments in different cranial nerves [8, 39, 41].

In 1904, Panse [32] was the first who described a case of a fibrillary astrocytoma of the VIII cranial nerve root entry zone, and Cushing [12] described a second case in 1917.

Arnautovic et al. were the first who reported a case of glioma from the proximal portion of the trigeminal nerve. Arnautovic et al. [2] concluded that most cases reported in the literature had been either fibrillary or gemistocytic astrocytomas [2, 3, 6, 15, 20, 21, 25]. Primary PA-CPA had been described by Francesco et al. [16] (a case of trigeminal nerve root entry zone PA) and Beutler et al. [3] (a case of VIII cranial nerve root entry zone PA). The first pediatric primary pilocytic astrocytoma had been reported by Mirone et al. [30].

In addition, malignant primary CPA gliomas had also been described so far. Breshears et al. [7] and Kasliwal et al. [23] described a case of a primary glioblastoma multiforme (GBM) of the trigeminal nerve root entry zone and primary GBM of the oculomotor nerve [34, 45], and the VIII cranial nerve [47] had also been reported.

Arnautovic [2] concluded that the longest glial segment is found in cranial nerve VIII and that this explained the predominance of cranial nerve VIII [22] as the origin of CPA glioma, followed by the sensory part of cranial nerve V, and then the facial and glossopharyngeal nerves.

### Secondary CPA glioma

Secondary CPA gliomas are exophytic tumors arising from the brainstem or the cerebellum with a secondary invasion of the CPA [16].

Choux et al. [11] described four different types of brainstem gliomas (type 1: diffuse brainstem gliomas; type 2 (solid or cystic): focal intrinsic tumors; type 3: exophytic tumors; type 4: cervicomedullary tumors).

Brainstem gliomas may show complex growth patterns [13] and typically rarely grow exophytically into the CPA [48].

In 1947, Revilla [35] published a series by Walter Dandy, who had operated on CPA tumors, and found 12 cases of gliomas of the CPA. Five cases were astrocytoma (but were not more precisely classified). In their study, Famer et al. [14] described brainstem gliomas of the pons with exophytic lateral growth (CPA glioma).

PA are typically intraparenchymal in origin and are rarely exophytic [2]. In the case of exophytic growth, they usually grow dorsally to the brainstem [26] and will largely fill the fourth ventricle [26], usually with little or no detectable infiltration of the brainstem.

In addition, other gliomas of the CPA (except PA) have been described in the literature. Non-pilocytic glioma of the CPA (NPG-CPA) included among other things anaplastic astrocytoma [24] and GBM [1, 29].

Matsuda et al. [29] summarized four cases [27, 46] (including their case) of exophytic GBM of the cerebellum with secondary invasion in the CPA, and also other authors [1, 10, 36] published cases of GBM of the CPA, in some cases even with secondary invasion of the cranial nerves by GBM [28, 33].

Other cases of CPA glioma, which are not more precisely classified, are described by Gentry et al. [17] (seven cases of CPA glioma out of 75 cases of CPA tumors) and Ozawa [31] (exophytic pontine glioma).

### PA-CPA glioma in children and adults

Guillamo et al. [19] concluded in their study, “adult brainstem gliomas are different from the childhood subtypes.” In adults, brainstem gliomas are, among other things, less aggressive than in children [19] (survival is significant shorter in children than in adults) [38]. They furthermore summarized that brainstem gliomas in adults are poorly understood because they are rarer in adults than they are in children [19].

Selvapandian et al. [38] concluded that the mean duration of symptoms before admission is significantly shorter in children (3.6 months in children versus 9.7 months in adults), while there was no significant difference in the clinical features. While the tumor grade seems to be a significant factor regarding the survival in adults, it seems not to be in children [38].

Exophytic gliomas account for 10–15% of brainstem gliomas in children [42].

Probably the largest series in the literature of PA-CPA had been published by Tomita and Grahovac [44], who analyzed CPA tumors in infants and children and found in a series of 44 CPA tumors, 11 were PA-CPA.

Tomita and Grahovac [44] discovered that from 12 benign secondary CPA glioma (11 PA and one ganglioglioma), 6 arise from the restiform body, two from the medulla oblongata, two from the pons, one from brachium pontis, and one from the cerebellum. However, they did not separate between the cerebellopontine angle and the cerebellomedullary fissure and included both of them in their study. Therefore, it is expected that some of the lesions are not strictly defined as CPA glioma.

In their series [44], 52% presented with hydrocephalus, while in the series of CPA lesions in children by Zuccaro and Sosa [49], 33% of patients presented with hydrocephalus. In our study, two of four patients had hydrocephalus.

Additional symptoms (gait ataxia, cranial nerve dysfunction, and facial weakness) were similar to our study.

One interesting finding in our study was that both patients with cystic PA-CPA were much younger when compared with both patients with solid PA-CPA (mean age 8.5 years versus 62 years).

Similar to Guillamo et al. [19] results, the differences in the mean ages and radiological findings suggest that these might be different subtypes of the disease.

In the series by Guillamo et al. [19] of 48 adult brainstem gliomas, 12 showed exophytic growth (10 prepontine and two posterior). However, the study included a very heterogeneous pathological spectrum (with only one PA), and the lateral growth in the CPA was not described.

To the best of our knowledge, the largest single institutional series of adult PA was published by Theeler et al. [43] (127 patients). They showed that the majority of adult PA are supratentorial [43]. The brainstem was involved in 24% of cases, and the cerebellum in 13% of cases [43].

### Surgical strategy

Even today, with new technical possibilities, surgery of pilocytic astrocytoma-cerebellopontine angle (PA-CPA) presents a formidable challenge. In 2004, Yousry et al. [48] demonstrated that specialized magnetic resonance imaging sequences (3D CISS and 3D MP-Rage) provided the surgeon preoperatively with important information for planning surgical management, such as the relationship between a tumor and adjacent neural structures (cranial nerves and brainstem) and the borders of the tumor within the cisterns.

In our opinion, also intraoperative monitoring (MEP and EMG recordings of the facial nerve and lower cranial nerves, and auditory evoked potentials, MEPs, and sensory evoked potentials of the upper and lower extremities) during surgery is of high importance and helps the surgeon to preserve cranial nerve functions.

Furthermore, it is essential to recognize that the surgical strategy in cystic and non-cystic PA-CPA is different. In the case of large cystic PA-CPA, a decompression of the cyst should be, in most cases, the first step (similar to the strategy described by Francesco et al. [16]) and the cystic wall should be removed when it is not too adherent to surrounding structures. After this step, the surface of the solid tumor portion should be incised, and debulking should be performed. The last step is to dissect the tumor from the surrounding structures. Arnautovic et al. concluded that usually in the case of PA-CPA, a clear arachnoid cleavage plane is found between the tumor and the surrounding tissue [2].

In the case of primary PA-CPA, there is a further important decision: Should one sacrifice or preserve the cranial nerve from which the tumor arose? In our study, we decided to sacrifice it, because the loss of cranial nerve VIII function on one side is compensable. Most authors, with similar cases of PA-CPA of cranial nerve VIII, made the same decision [3, 30, 45]. The decision would be more complicated in the case of the PA originating from cranial nerve VII. In such a case, partial resection or gross total removal with facial nerve anastomosis using a sural nerve graft should be discussed.

In a case of secondary PA-CPA, the extent of resection depends on the infiltration of the brainstem, the cerebellar peduncle, and the cerebellum.

In our study, three CPA gliomas were totally removed, and one CPA glioma was subtotally removed. At the same time, the facial nerve was anatomically preserved in all cases and showed good functional outcome in all cases in the long term. The patient with the incomplete resection of the PA-CPA demonstrated a slight progression of residual tumor tissue 1 year after the resection. On further follow-up, no further progression was observed.

In a case of clear further progression or recurrence, a future second surgery, radiotherapy, or chemotherapy should be discussed, in our opinion (according to our tumor board).

Similar to our results, most authors concluded that, in cases of exophytic brain stem gliomas, a gross total or subtotal removal with a favorable prognosis is possible in most patients [18].

Regarding the clinical outcome and complications, it is difficult to find clear analyses in the literature. While primary PA-CPA are only described in single case reports, secondary PA-CPA are only described as part of other gliomas of CPA or PA of other locations (usually, most studies did not separate PA-CPA from PA of the brainstem, but especially for the clinical outcome, this is an essential difference).

The series by Walter Dandy [35] focused on the differential diagnosis of gliomas of the CPA from other lesions of the CPA. However, no outcome is mentioned, and only astrocytomas of CPA (without any more precisely histopathological classification) are described.

Farmer et al. [14] focused on brainstem gliomas in common, but one group concentrated on brainstem gliomas of the pons with exophytic lateral growth (CPA glioma). They concluded [14] that in cases of non-diffuse brainstem tumors, 1994 marked a pivotal year in their treatment. While before 1994, 75% of patients received biopsy, but in the time after 1994, only 15% received biopsy, as radical surgery became the treatment of choice.

To best of our knowledge, there is no study about PA-CPA in literature.

### Limitations

The main limitation of this study is the small number of patients, due to the rare prevalence of PA-CPA.

In summary, CPA gliomas can be classified into primary and secondary CPA gliomas depending on their site of origin.

Two different subtypes of PA-CPA exist: (1) cystic PA-CPA in young patients and (2) non-cystic PA-CPA in older patients.

A PA-CPA can be safely removed, and most complications that occur immediately after surgery have resolved in the long-term follow-up. Nevertheless, the treatment of such a rare tumor remains a challenge.
